# PPARγ Regulates Expression of Carbohydrate Sulfotransferase 11 (*CHST11/C4ST1*), a Regulator of LPL Cell Surface Binding

**DOI:** 10.1371/journal.pone.0064284

**Published:** 2013-05-16

**Authors:** Ismayil Tasdelen, Ruud Berger, Eric Kalkhoven

**Affiliations:** Department of Metabolic Diseases and The Netherlands Metabolomics Center, University Medical Centre Utrecht, Utrecht, The Netherlands; Universite de Sherbrooke, Canada

## Abstract

The transcription factor PPARγ is the key regulator of adipocyte differentiation, function and maintenance, and the cellular target of the insulin-sensitizing thiazolidinediones. Identification and functional characterization of genes regulated by PPARγ will therefore lead to a better understanding of adipocyte biology and may also contribute to the development of new anti-diabetic drugs. Here, we report carbohydrate sulfotransferase 11 (Chst11/C4st1) as a novel PPARγ target gene. Chst11 can sulphate chondroitin, a major glycosaminoglycan involved in development and disease. The Chst11 gene contains two functional intronic PPARγ binding sites, and is up-regulated at the mRNA and protein level during 3T3-L1 adipogenesis. Chst11 knockdown reduced intracellular lipid accumulation in mature adipocytes, which is due to a lowered activity of lipoprotein lipase, which may associate with the adipocyte cell surface through Chst11-mediated sulfation of chondroitin, rather than impaired adipogenesis. Besides directly inducing Lpl expression, PPARγ may therefore control lipid accumulation by elevating the levels of Chst11-mediated proteoglycan sulfation and thereby increasing the binding capacity for Lpl on the adipocyte cell surface.

## Introduction

The close connections between obesity and its complications, such as type 2 diabetes and cardiovascular diseases, has firmly established white adipose tissue as a key regulator of whole body glucose and lipid metabolism [1]. White adipose tissue mainly regulates metabolism through storage of lipids (as triglycerides) and the secretion of so-called adipokines, which function in an endocrine or paracrine fashion. Several independent lines of research have firmly established the transcription factor peroxisome proliferator activator γ (PPARy) as the master regulator of adipocyte differentiation, maintenance and function. For example, *in vitro* differentiation of fibroblasts into mature white adipocytes can be induced by introduction of PPARγ [2]. In addition, this protein directly regulates a large set of “adipocyte genes” involved in lipid and glucose metabolism [3], [4]. Furthermore, PPARγ^−/−^ mice are severely lipodystrophic, while PPAR γ^+/−^ mice have reduced amounts of adipose tissue [5], [6], [7], [8]. PPARγ is also essential for the maintenance of adipose tissue, since conditional knockout of the *Pparg* gene resulted in reduced *in vivo* survival of mature adipocytes [9]. Finally, human Familial partial lipodystrophy subtype 3 (FPLD3, MIM 604367) patients, harbouring heterozygous mutations in the *PPARG* gene, are characterized by aberrant fat distribution and metabolic disturbances, including insulin resistance and dyslipidaemia [10].

PPARγ activity can be stimulated by thiazolidinediones (TZDs), a class of anti-diabetic drugs that includes rosiglitazone [11]. Since elevated levels of serum free fatty acids promote insulin resistance [12], an important potential mechanism for the beneficial effects of TZDs is therefore the net partitioning of lipids in adipose tissue. This may partly be explained by the PPARγ-mediated stimulatory effect of TZDs on adipocyte differentiation, resulting in increased lipid storage capacity in adipose tissue. In addition, PPARγ also directly regulates genes involved in all different aspects of lipid handling, such as lipid uptake (e.g. lipoprotein lipase (Lpl) [13]), intracellular lipid transport (e.g. fatty acid binding protein 4 (Fabp4) [14]) and lipid storage (e.g. perilipin [15]), as well as anti-lipolytic genes (e.g. GPR81 [16]). While PPARγ is clearly a suitable pharmacological target, TZD use has unfortunately been linked to adverse side effects such as undesired weight gain, fluid retention, peripheral oedema, and potential increased risk of cardiac failure [11], [17]. Interestingly, recent findings indicate that a more restricted modulation of PPARγ activity may provide a new way of improving insulin sensitivity. A clear example of this is the recently identified phosphorylation site at serine 273 in PPARγ. CDK5-mediated phosphorylation of serine 273 in PPARγ leads to deregulation of a subset of genes whose expression is altered in obesity including the insulin-sensitizing adipokine, adiponectin [18]. Interestingly, S273 phosphorylation is blocked *in vivo* and *in vitro* by TZDs, but also by certain antidiabetic drugs that are weak PPARγ agonists or non-agonists [18], [19]. These findings indicate that a comprehensive view on the mechanisms regulating PPARγ activity as well as its downstream target genes is required to develop the next generation of PPARγ-based antidiabetic drugs.

In the past few years, several genome wide PPARγ binding profiles have been generated in adipocytes, using either ChIP-ChIP [4], [20], [21], ChIPseq [3], [22] or ChIP-PET technology [23]. These global approaches have provided important new concepts, like the extensive crosstalk between PPARγ and C/EBPα as deduced from the overlap in their cistromes [3], [4]. The binding profiles have also given important information on the single gene level, i.e. the identification of novel PPARγ target genes involved in lipid and glucose metabolism (e.g. Agpat2 and Hk2, respectively; [3]). Furthermore, genome wide binding profiles have helped to elucidate complex gene regulatory mechanisms, as exemplified by the genomic GPR81-GPR109A and UCP3-UCP2 regions, where single PPREs regulate multiple genes [16], [24].

Using the PPARγ-RXR ChIPseq profile by Nielsen *et al.* [3] as a starting point, we identified the mouse chrondroitin-4-sulfotransferase 1 gene (*C4ST1/Chst11*) as a novel target of PPARy. Chst11 is a Golgi-bound enzyme that catalyses the transfer of sulphate groups to the 4-O position of chondroitin sulphate (CS) and dermatan sulphate (DS). Membrane-bound sulphated proteoglycans are necessary for lipid accumulation in adipocytes, possibly because of their ability to interact with lipases like Lpl [25], [26]. We found Chst11 mRNA and protein expression to be upregulated by PPARy during 3T3-L1 adipogenesis and identified two functional intronic PPARy binding sites in the Chst11 gene. In common with disruption of Lpl function [27], siRNA-mediated knock down of Chst11 resulted in reduced intracellular lipid accumulation in mature 3T3-L1 adipocytes. This effect is probably not due to inhibition of adipogenesis, as the expression of typical adipogenic genes such as *C/EBPa*, *Fabp4*, *Lpl* and adiponectin (*Adipoq*) was unaffected. Rather, knockdown of Chst11 inhibited the activity of Lpl. These findings suggest that PPARy may regulate Lpl-mediated lipid accumulation by two different mechanisms: it increases *Lpl* transcription directly [13], but can also indirectly regulate activity of the Lpl protein by elevating Chst11 expression and thereby increasing the number of docking sites for Lpl on the adipocyte cell surface.

## Materials and Methods

### Materials

Rosiglitazone and GW9662 were purchased from Alexis Biochemicals and Cayman Chemical, respectively. Heparin was purchased from LEO Pharma. Oil-red-o, dexamethasone and IBMX were from Sigma Aldrich. The Lpl activity kit was from Roar Biomedical. Anti-PPARγ (sc-7196) from Santa Cruz Biotechnology was used for ChIP assays. Anti-PPARγ (sc-7273) antibody from Santa Cruz Biotechnology and anti-Chst11 (ab57225) antibody from Abcam were used for immunofluorescence.

### Plasmids

The regions surrounding peak 1 and 2 in the intron of the Chst11 gene were subcloned using the following primers. Peak 1, forward: 5′-CGGGGTACCCCGTGAAGACTAAGATAACATAG-3′ and reverse: 5′-CCGCTCGAGCGGACACACACACACATTCAGCTC-3′; Peak 2, forward: 5′-CGGGGTACCCCG TTCCCGCTCTGGGAAAAAAAG-3′ and reverse: 5′-CCGCTCGAGCGGGACCTGGTCTTCCCTGTCTTGATG-3′. The products were cut with KpnI and XhoI and ligated into the TK-Pgl3 vector [28]. All other plasmids have been described before [29], [30].

### Cell Culture, Differentiation and Reporter Assays

The human osteosarcoma cell line U2OS (ATCC, Manassas, VA) was maintained in DMEM Glutamax (Dulbecco) containing 10% foetal calf serum (Life Technologies, Inc., Rockville, MD), penicillin and streptomycin (both 100 µg/ml; Life Technologies). The murine 3T3-L1 cell line (ATCC, Manassas, VA) was cultured in the same media but with 10% bovine serum (Life Technologies) and penicillin and streptomycin (both 100 /ml; Life Technologies). For differentiation, 3T3-L1 cells were grown to confluence and after 2 days incubated with culture medium containing dexamethasone (250 nM), 3-isobutyl-1-methylxanthine (500 µM) and insulin (170 nM) for 2 days. On day 3, medium was changed for culture medium supplemented with insulin (170 nM) and left for a week. Subsequently, cells were stained with Oil-red-O, or lysed and subjected to Western blot analysis as described before at day 5 after differentiation [16], [29], [30]. For Western blot analyses, differentiated 3T3-L1 cells were lysed in RIPA lysis buffer (200 mm Tris-HCl, pH 8.0; 0,1% SDS, 1% Triton X-100; 10 mm EDTA; 150 mm NaCl; 1% sodium deoxycholate containing protease inhibitors). Cell lysates were subjected to SDS-PAGE, and transferred to Immobilon membranes (Millipore). ECL Plus (PerkinElmer Life Sciences) was used for detection on an ImageQuant LAS 4000 (GE Lifesciences).

Reporter assays were performed essentially as described before [29], [30]. In short, cells were transfected in 24-well plates with 1 µg reporter plasmid, 10 ng PPARy expression construct, and 2 ng pCMV-Renilla reporter plasmid (Promega). The next day, cells were washed twice with PBS and subsequently maintained in medium in absence or presence of rosiglitazone for 24 h. After incubation, cells were washed twice with PBS and harvested in passive lysisbuffer (Promega) and assayed for luciferase activity according to the manufacturer’s protocol (Promega Dual-Luciferase Reporter Assay System) and for Renilla luciferase activity to correct for transfection efficiency. The relative light units were measured by a CentroLB 960 luminometer (Berthold Technologies, Bad Wildbad, Germany).

### Chromatin Immunoprecipitation (ChIP)

Chromatin immunoprecipitation (ChIP) was performed exactly as decribed before [30]. Quantitative PCR was performed with primers against mouse Fabp4 PPRE (5′-GAGAGCAAATGGAGTTCCCAGA-3′; 5′-TTGGGCTGTGACACTTCCAC-3′), Chst11 peak 1 (5′-ACAGGCTTGCTTTGGCAC-3′; 5′-ACACTCACTACTCACAATCTGT-3′), Chst11 peak 2 (5′-CTCATCCAACCTGGGTTTTGG-3′; 5′-GAGTTCCTAGACTTGAAGAACTATG-3′) and the mouse beta globin gene as control (5′-CCTGCCCTCTCTATCCTGTG-3′; 5′- GCAAATGTGTTGCCAAAAAG-3′).

### siRNA Transfection

3T3-L1 cells were transfected with siRNA oligonucleotides as described before [30] using Lipofectamine RNAiMax (Invitrogen) according to the manufacturer’s protocol. The siRNA oligonucleotides used were siControl (D-001810-10-20, Dharmacon), siPPARγ (L-040712-00-0010, Dharmacon), siChst11 (J-040396-11-0020, Dharmacon), siLpl (L-042649-01-0005, Dharmacon).

### RNA Isolation and qPCR

3T3-L1 fibroblasts were differentiated as described above. Four independent samples of total RNA were extracted at different time points using TRIzol reagent (Invitrogen). cDNA was synthesized using the superscript first strand synthesis system (Invitrogen) according to manufacturer’s protocol. Gene expression levels were determined by quantitative real time PCR with the MyIq cycler (Bio-Rad) using SYBR-green (Bio-Rad) and normalized to *TFIIb* expression.

The primers used were as follows: murine TFIIb forward primer, 5′-TCCTCCTCAGACCGCTTTT-3′, and reverse primer, 5′-CCTGGTTCATCATCGCTAATC-3′; murine PPARy forward primer, 5′-CGCTGATGCACTGCCTATGA-3′ and reverse primer, 5′-AGAGGTCCACAGAGCTGATTCC-3′; murine Chst11 forward primer, 5′-GTCCCCTGCAGGAGCTCTA-3′, and reverse primer, 5′-CTCATCTGGTGCAGGATGG-3′; murine Fapb4 forward primer, 5′-CGCAGACGACAGGAAGGT-3′, and reverse primer, 5′-TTCCATCCCACTTCTGCAC-3′; murine Adipoq forward primer, 5′-GGAACTTGTGCAGGTTGGAT-3′, and reverse primer, 5′- TCTCCAGGCTCTCCTTTCCT-3′; murine Lpl forward primer, 5′- TTTGTGAAATGCCATGACAAG-3′ and reverse primer, 5′-TCAAACACCCAAACAAGGGTA-3′; murine C/EBPα forward primer, 5′-AAACAACGCAACGTGGAGA-3′ and reverse primer, 5′-GCGGTCATTGTCACTGGTC-3′.

### Immunofluorescence

For immunofluorescence staining, 3T3-L1 cells were plated on glass coverslips and differentiated for 5 days. Subsequently, cells were fixed with 4% paraformaldehyde (20′, RT) and permeabilized in PBS supplemented with 0.2% Triton for 5 minutes. After 30′ incubation in blocking buffer (2% BSA in PBS), cells were stained with primary antibodies for 2 h at room temperature, and then incubated with secondary fluorochrome-conjugated antibodies. After several washes, coverslips were incubated Nile-Red and Hoechst, washed with PBS, mounted in Mowiol and analysed with an LSM710 Met confocal microscope (Carl Zeiss, Jena, Germany).

### Lpl Activity Assays

Lpl activity was measured according to the manufacturers instructions. In short, cells were grown and differentiated in 6-well plates. Media were removed and cells were incubated with 50 units of heparin in 500 ul of PBS (30′, 37**°**C). Supernatants were collected and debris was removed by centrifugation (12.000 rpm, 10′). Supernatants (25 µdebris was removed by cassay buffer mix (175 µssay buff’ ανδ analysed on a Victor3 (Perkin Elmer) at wavelengths 355/460.

## Results

### Chst11 is a Novel PPARy Target Gene

**Figure 1:**
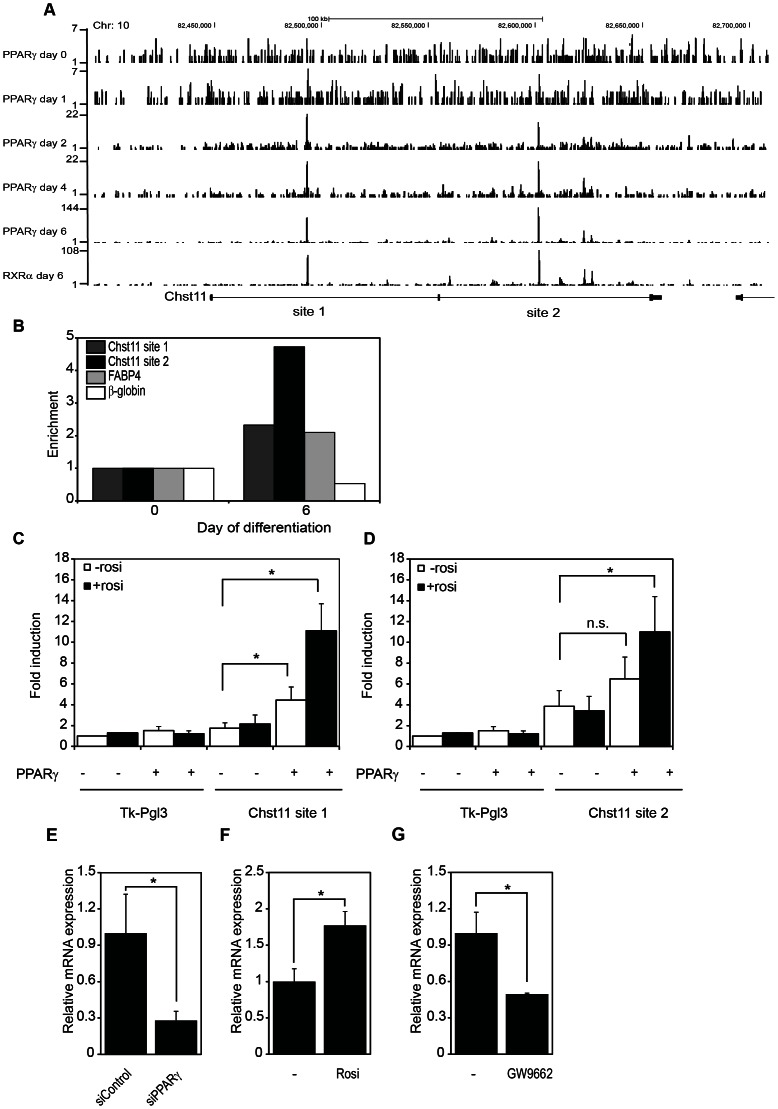
Chst11 is a novel PPARy target gene. (A) Chip-Seq data of PPARγ and RXRα occupancy on the Chst11 gene according to Nielssen et al. [3]. UCSC Genome Browser tracks at day 0, 1, 2 4 and 6 of differentiation are shown. Please note differences in y-axes. Two intronic PPARy-RXRα binding sites were designated site 1 and 2. (B) ChIP-PCR on 3T3-L1 preadipocytes and adipocytes. Chromatin was prepared on day 0 and day 6 of differentiation and subjected to immunoprecipitation using antibodies against PPARγ. Enriched DNA was analysed using quantitative PCR with primers located at site 1 and 2 in the Chst11 gene (dark gray and blacks bars, respectively). As a positive control, primers located at the Fabp4 PPREs (∼5500 bp from transcription start site; light gray bars) were used, primers located in the globin locus were used as a negative control (white bars). Results are shown relative to normalized ChIP recovery data of day 0 and results are representative of at least 3 independent experiments. (C and D) U2OS cells were cotransfected with a reporter construct (TKpGL3) containing Chst11 site 1 or site 2 sequences, or the parental reporter construct, together with empty (pCDNA) or PPARγ encoding expression vectors. Activation of the luciferase reporter in the absence or presence of 1 µM rosiglitazone is expressed as fold induction over that with empty reporter cotransfected with pCDNA in the absence of rosiglitazone after normalisation for Renilla luciferase activity. The error bars display SEM and significance is shown by the asterisks (p<0.05), n = 3 (E) Chst11 mRNA expression in 3T3-L1 adipocytes that had been treated with control or PPARy siRNA from the start of differentiation and analyzed at day 5. Relative mRNA expression levels were related to control siRNA treated cells and normalized for the TFIIb reference gene. The error bars display SEM and significance is shown by the asterisks (p<0.05), n = 3 (F) Chst11 mRNA expression in 3T3-L1 adipocytes and the effect of rosiglitazone treatment (1 µM, 24 h). Relative mRNA expression levels were normalized for the TFIIb reference gene. (G) As in panel F, but after treatment with the PPARγ antagonist GW9662 (10 µM, 24 h).

To identify novel PPARy target genes we thoroughly analysed ChIPseq data generated by Nielsen *et al.* [3]. We focused on loci that displayed PPARy/RXRα binding at day 6 with little or no binding of these transcription factors at day 0 of 3T3-L1 adipogenesis. Using these criteria we identified the carbohydrate sulfotransferase 11 *Chst11*, also known as C4st1, as a potential direct PPARγ target gene. Chst11 can sulphate chondroitin sulphate-proteoglycans (CSPG), which plays an important role in development and disease ([31], [32]; see also Discussion). Two intronic binding sites for PPARy were observed on day 2 of adipogenesis, with increased binding during later stages of adipogenesis (day 4 and 6; Fig. 1A). These binding sites were also present in the binding profiles of RXRα, which is required for PPARy to bind to DNA. Please note that these sites do not overlap with the regulatory elements previously identified in the Chst11 locus, some of which confer regulation by TGFβ [33].

The recruitment of PPARγ to the two intronic binding sites in the Chst11 gene in mature adipocytes (day 6) was confirmed by ChIP-PCR (Fig. 1B). As a positive control the well-characterized PPARy/RXRα enhancer bindingsite in the Fabp4 gene was used. In addition, the recruitment in preadipocytes (day 0), in which PPARγ expression is low, was negligible and PPARγ was not detected on an arbitrary region of the beta-globin gene, which served as a negative control (Fig. 1B). Having established that PPARy is recruited to site 1 and 2 upon differentiation (Fig. 1A and B), we wished to investigate whether these intronic binding sites could act as functional enhancers. Therefore, both regions were cloned in a promoter-containing reporter plasmid (TK-pGL3). The activity of these reporters was determined in human osteosarcoma (U2OS) cells, which express negligible levels of endogenous PPARγ protein, but display a robust response upon overexpression of the protein [29]. As shown in Figure 1C and D, transfection of cells with an expression vector encoding PPARγ2 activated the reporter genes containing PPARy/RXRα binding sites of the Chst11 gene compared to empty vector control (pCDNA), most markedly for binding site 1. Activation of PPARγ by the synthetic ligand rosiglitazone further enhanced the PPARγ-mediated activation of both reporters (Fig. 1C and D), indicating that the two intronic PPARy/RXRα binding sites in the Chst11 gene can act as functional enhancers.

To corroborate these findings, we investigated whether modulation of endogenous PPARγ protein affected Chst11 expression. First, siRNA-mediated knock down of PPARγ was performed. PPARγ knock down resulted in a significant reduction of Chst11 mRNA expression in mature adipocytes (Fig. 1E). Furthermore, treatment of mature adipocytes with the PPARγ agonist rosiglitazone resulted in increased Chst11 levels (Fig. 1F), while incubation with the antagonist GW9662 lowered Chst11 expression (Fig. 1G). Taken together, these data classify Chst11 as a novel direct PPARγ target gene.

### Chst11 is Expressed in Mature Adipocytes

**Figure 2:**
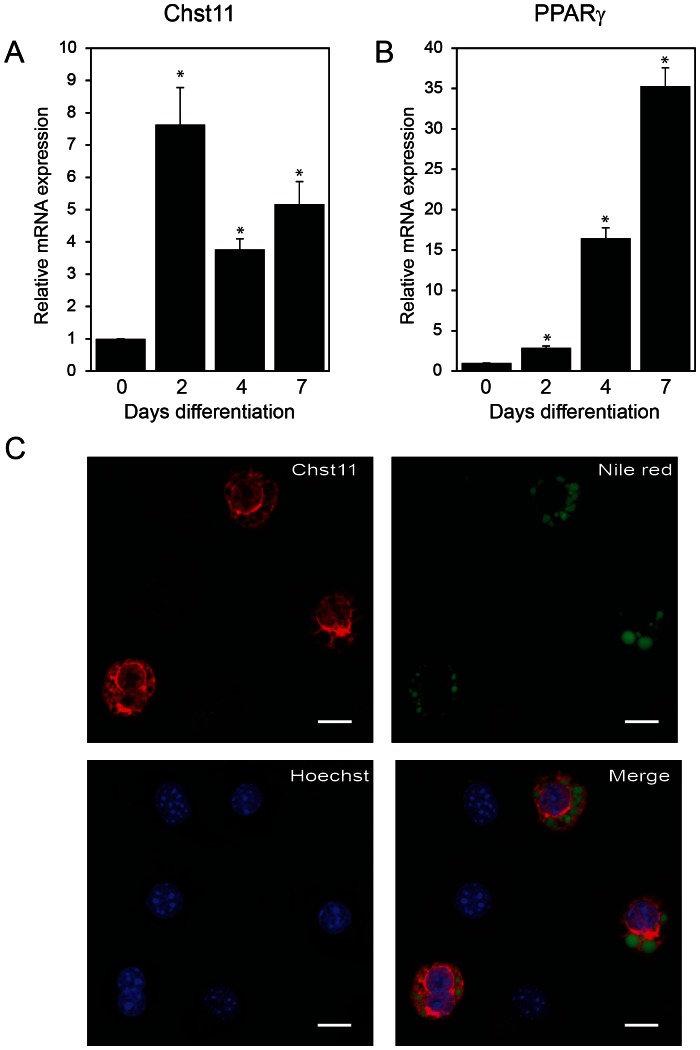
Chst11 is expressed in mature 3T3-L1 adipocytes. (A) and (B): mRNA expression profiles of Chst11 and PPARy in adipogenesis of 3T3-L1 cells during different days. Data are represented as described in Figure 1E. (C) Representative confocal microscopy images of 3T3-L1 (pre)adipocytes. Endogenous Chst11 (red) was visualized utilizing specific antibodies, differentiated cells were identified with Nile Red (green), Hoechst was used to visualize the nuclei (blue).

Next, we examined Chst11 mRNA and protein expression in adipogenesis. Using quantitative RT-PCR, Chst11 mRNA levels were found to increase 8 fold and reached its climax at day 2 of differentiation after which the levels were stable (Fig. 2A). As a control, expression of PPARγ was analysed, which increased steadily over the course of differentiation (Fig. 2B). To investigate Chst11 protein expression and localization, immunofluorescent staining of Chst11 was combined with fluorescent staining for intracellular lipid droplets (Nile red) to mark differentiated cells. As shown in Fig. 2C, mature adipocytes (Nile Red positive) also stained positive for Chst11 protein, while Chst11 could not be detected in preadipocytes (Nile Red negative). Chst11 displayed cytoplasmic localization in mature adipocytes, in agreement with other cell types [31]. In summary, these data indicate that Chst11 is induced during adipogenesis, both at the mRNA and protein level.

### Knockdown of Chst11 Leads to Decreased Intracellular Lipid Accumulation, but not Adipogenesis

**Figure 3:**
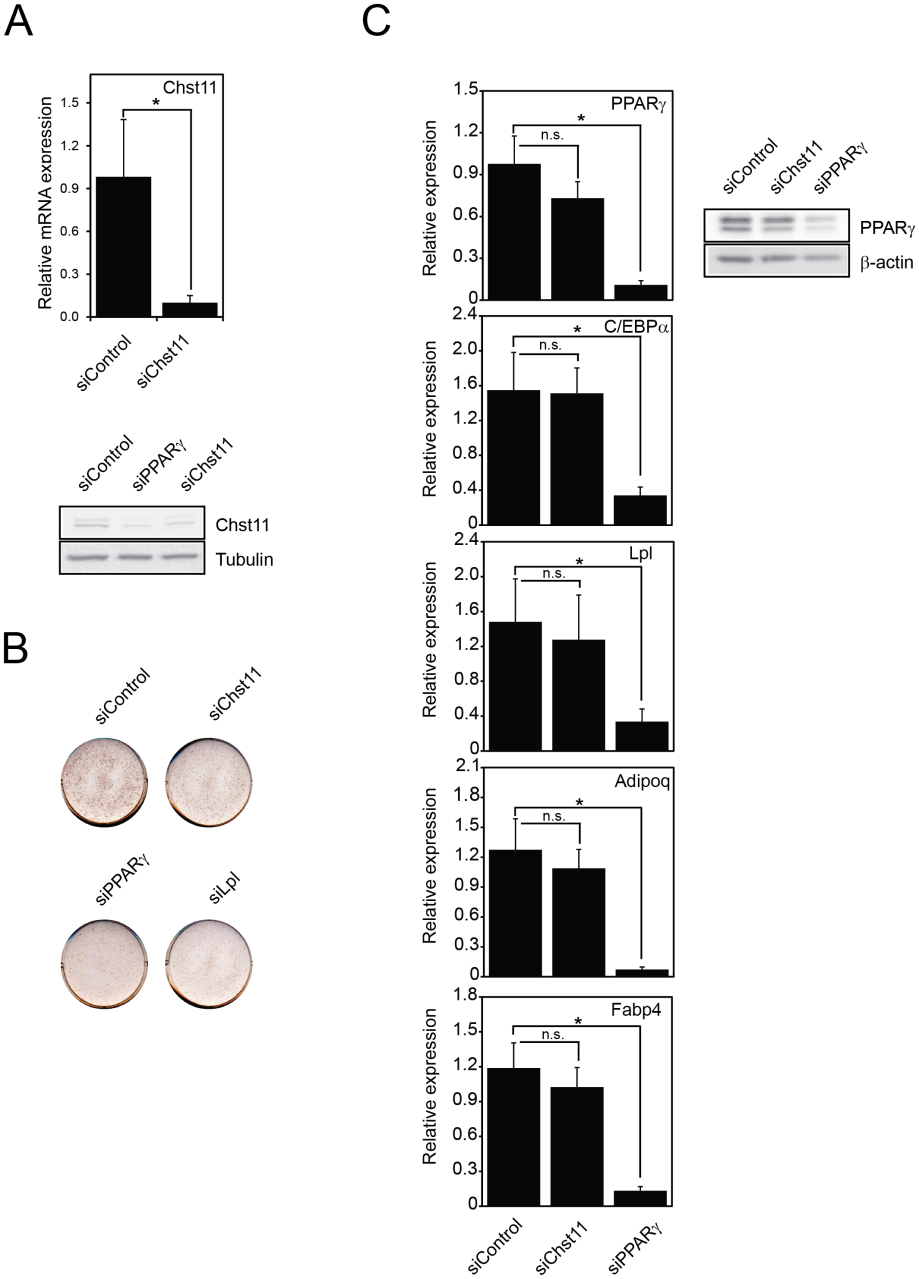
Knockdown of Chst11 leads to decreased lipid accumulation but not adipogenesis. (A) 3T3-L1 cells were treated with siRNA oligonucleotides targeting Chst11 or control (scrambled) oligonucleotides and Chst11 mRNA and protein expression levels were determined at day 5 after differentiation by qPCR (upper panel) and Western blotting (lower panel), respectively. The error bars display SEM and significance is shown by the asterisks (p<0.05), n = 3 (B) 3T3-L1 cells were treated with siRNA oligonucleotides targeting PPARy, Chst11, Lpl, or control (scrambled) oligonucleotides, fixed and stained for lipid accumulation using Oil-red-O. Pictures are representative of 3 independent experiments. (C) 3T3-L1 cells were treated with siRNA oligonucleotides targeting Chst11, PPARy or control (scrambled) oligonucleotides and mRNA expression levels of PPARγ, C/EBPα, Lpl, adiponectin (adipoq) and Fabp4 were determined after 5 days of differentiation. Relative mRNA expression levels were related to control siRNA treated cells and normalized for the TFIIb reference gene. Samples were also generated for a western blot in which PPARγ expression was shown upon Chst11 and PPARγ knockdown. The error bars display SEM and significance is shown by the asterisks (p<0.05), n = 3.

Having established that PPARy regulates Chst11 levels (Fig. 1) and that Chst11 is expressed on the mRNA and protein level in mature adipocytes (Fig. 2), we wished to investigate the role of Chst11 in adipocytes. To address this, siRNA-mediated knockdown was performed in 3T3-L1 cells, and cells were subsequently subjected to adipogenic culture conditions. siRNA-mediated knockdown reduced Chst11 mRNA and protein (Fig 3A). Chst11 knock down led to decreased lipid accumulation, as assessed by Oil-red-O staining of intracellular lipids, a phenomenon also observed upon Lpl knock down (Fig. 3B) [27]. Reduced lipid staining may be the result of reduced adipogenesis or only reduced lipid accumulation. To distinguish between these possibilities the effect of Chst11 knock down on the expression of *C/EBPα* and *PPARγ*, two key regulators of adipogenesis, was examined. Only marginal differences were observed between cells that had been treated with Chst11 siRNA oligonucleotides or scrambled siRNA oligonucleotides (Fig. 3C). PPARy protein levels were also not affected dramatically by Chst11 knockdown. Expression of the genes encoding Lpl, adiponectin (Adipoq) and FABP4, which are all up-regulated in adipogenesis, was also unaffected by Chst11 knock down. Knock down of PPARγ, which has repeatedly been reported to block adipogenesis (e.g. [8] [34]), however significantly inhibited the expression of all these genes. These findings therefore indicate that Chst11 specifically regulates lipid accumulation rather than adipogenesis.

### Knockdown of Chst11 Leads to Decreased Lpl Activity

**Figure 4:**
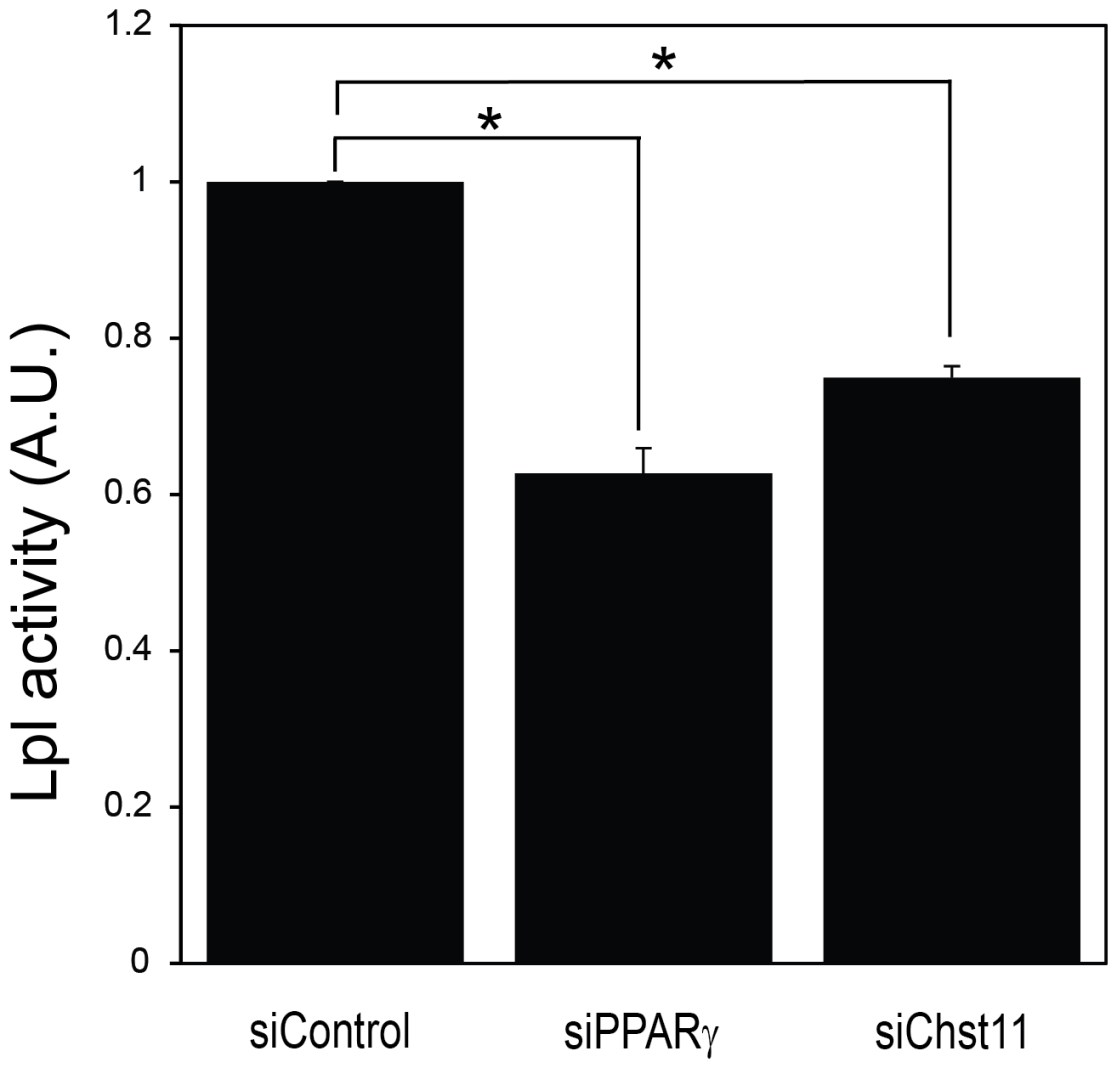
Knockdown of Chst11 leads to decreased Lpl activity. 3T3-L1 cells were treated with siRNA oligonucleotides targeting Chst11, PPARy or control (scrambled) oligonucleotides and Lpl activity was analysed. The error bars display SEM and significance is shown by the asterisks (p<0.05), n = 3.

Since Chst11 knock down reduced lipid accumulation (Fig. 3B) and given that Lpl is required for lipid accumulation in 3T3-L1 adipocytes (Fig. 3B) [27] we hypothesized that Chst11 activity may regulate Lpl activity in mature adipocytes. In favour of this hypothesis is the finding that Lpl requires a stable negatively charged docking site on HSPGs for binding to the cell surface of endothelial cells [35], which can be provided by Chst11-mediated sulfation of CS in adipocytes. To test this possibility, 3T3-L1 cells were differentiated and subjected to scrambled, PPARγ and Chst11 siRNA-mediated knock down, after which Lpl was released from the adipocyte cell surface by heparin treatment [36], and enzymatic activity was determined as a measure for cell-surface-associated Lpl. Upon knock down of Chst11 a significant reduction in Lpl activity was observed (Fig. 4; p<0.05). As a control, knock down of PPARγ, which regulates the expression of both Chst11 (current study) and Lpl [13], was performed. As expected, reduced PPARγ expression also resulted in impaired Lpl activity (Fig. 4). These data support a model in which PPARy regulates Chst11 expression, leading to increased sulphated CS chains which can then form docking sites for Lpl, ultimately contributing to lipid accumulation in mature adipocytes.

## Discussion

Multiple extracellular signalling pathways have been implicated in adipogenesis, ultimately leading to upregulation of the transcription factor PPARy, the master regulator of adipogenesis [37]. PPARy directly regulates the expression of genes involved in various aspects of lipid handling, including Lpl [38]. Here we have identified the enzyme Chst11, which harbours sulfotransferase activity, as a novel PPARγ target gene. Knockdown of Chst11 does not affect the adipogenic gene program, but rather inhibits lipid accumulation, possibly through inhibition of the cell surface binding of Lpl. These findings suggest that the central role of PPARy in lipid metabolism in adipocytes extends beyond the regulation of genes directly involved in lipid handling, and includes genes like Chst11, which play a more indirect role. Chst11 can sulphate the 4-O position of GalNac residues in chondroitin sulphate (CS), one of the major classes of glycosaminoglycans (GAG) [39], [40]. GAGs are sulphated repeating disaccharide units, which together with core proteins can form proteoglycans like heparan- and chondroitin sulphate proteoglycan (HSPG/CSPG), and function in cell-cell communication, adhesion, and protein presentation [41]. In addition, CSPG/HSPG are necessary for lipid accumulation in adipocytes [25]. Sulfation of GAGs by sulfotranferases like Chst11 adds negative charge to proteoglycans, which is important for interactions with various other proteins like growth factors, apolipoproteins, extracellular matrix, and plasma proteins [41]. Importantly, sulphated and thus negatively charged HSPG can function as a docking site for Lpl in endothelial cells [35], [42]. Furthermore, macrophages produce an oversulfated CSPG that can also bind Lpl [43], and CSPG is the dominant proteoglycan on the adipocyte cell surface [25], [44]. When combined with our current data, these findings suggest that the main role of Chst11 in adipocytes may be to increase the amount of sulphated, negatively charged CS chains that could then act as binding sites for Lpl activity on the adipocyte cell surface. Additional experiments are required to establish the sulphation status of CSPG, and its role in Lpl binding in adipocytes.

Lpl is best known for its role in endothelium, where secreted Lpl travels through the capillary walls towards the luminal space of the endothelium, and binds to the proteoglycans that are anchored into vascular endothelial cells [42], [45], [46], [47]. The transport of Lpl from adipocytes and myocytes, which produce high Lpl levels, to the capillary walls has just recently been unravelled. The GPIHBP1 protein, which is present on both apical and basolateral surface of endothelial cells, is capable of transporting Lpl from basolateral to the apical surface of the cells [48]. At the luminal side, Lpl can help to cleave lipids off chylomicrons and VLDL particles, so that the fatty acids can enter through the capillaries towards the adipocytes where it can be stored. Lpl molecules that are internalized by endothelial cells are recycled back to the cell surface [26]. Although Lpl binds to the surface of adipocytes with 5–10 fold higher efficiency compared to endothelial cells [49], its role in adipocytes is less well-defined. Multiple studies have provided evidence that lipolysis of lipoproteins at the endothelium by Lpl can loosen the junctions between endothelial cells so that large lipid enriched lipoproteins can pass through them [45], [50], [51]. In this event, Lpl should also be present at the cell surface of adipocytes to deplete the lipoproteins of their lipids. Surface-bound Lpl activity, which may be positively regulated by sulfotransferases like Chst11, can therefore be regarded as a back-up system for efficient take-up of lipids from lipoproteins. As genetic inactivation of Chst11 is associated with severe developmental abnormalities [31], adipose tissue-specific Chst11 knock-out mice will need to be generated to establish the precise role for Chst11 in lipid accumulation in adipocytes *in vivo*.
